# Cell Culture Platforms with Controllable Stiffness for Chick Embryonic Cardiomyocytes

**DOI:** 10.3390/biomimetics4020033

**Published:** 2019-04-27

**Authors:** María Luisa Durán-Pastén, Daniela Cortes, Alan E. Valencia-Amaya, Santiago King, Gertrudis Hortensia González-Gómez, Mathieu Hautefeuille

**Affiliations:** 1 Taller de Biofísica de Sistemas Excitables, Facultad de Ciencias, Universidad Nacional Autónoma de México, 04510 México City, Mexico; daniela_cg@ciencias.unam.mx (D.C.); valenciamaya@ciencias.unam.mx (A.E.V.-A.); santiagoking@ciencias.unam.mx (S.K.); hortecgg@ciencias.unam.mx (G.H.G.-G.); 2 Laboratorio Nacional de Canalopatias LaNCa, Instituto de Fisiología Celular, Universidad Nacional Autónoma de México, 04510 México City, Mexico; 3 Laboratorio Nacional de Soluciones Biomiméticas para Diagnóstico y Terapia LaNSBioDyT, Facultad de Ciencias, Universidad Nacional Autónoma de México, 04510 México City, Mexico; mathieu_h@ciencias.unam.mx; 4 Departamento de Física. Facultad de Ciencias Universidad Nacional Autónoma de México; 04510 México City, Mexico

**Keywords:** microscaffolds, cardiomyocytes culture, intracellular Ca^2+^ signals, stiffness, PDMS

## Abstract

For several years, cell culture techniques have been physiologically relevant to understand living organisms both structurally and functionally, aiming at preserving as carefully as possible the in vivo integrity and function of the cells. However, when studying cardiac cells, glass or plastic Petri dishes and culture-coated plates lack important cues that do not allow to maintain the desired phenotype, especially for primary cell culture. In this work, we show that microscaffolds made with polydimethylsiloxane (PDMS) enable modulating the stiffness of the surface of the culture substrate and this originates different patterns of adhesion, self-organization, and synchronized or propagated activity in the culture of chick embryonic cardiomyocytes. Thanks to the calcium imaging technique, we found that the substrate stiffness affected cardiomyocyte adhesion, as well as the calcium signal propagation in the formed tissue. The patterns of activity shown by the calcium fluorescence variations are reliable clues of the functional organization achieved by the cell layers. We found that PDMS substrates with a stiffness of 25 kPa did not allow the formation of cell layers and therefore the optimal propagation of the intracellular calcium signals, while softer PDMS substrates with Young’s modulus within the physiological in vivo reported range did permit synchronized and coordinated contractility and intracellular calcium activity. This type of methodology allows us to study phenomena such as arrhythmias. For example, the occurrence of synchronized activity or rotors that can initiate or maintain cardiac arrhythmias can be reproduced on different substrates for study, so that replacement tissues or patches can be better designed.

## 1. Introduction

Cardiomyocyte isolation and culture techniques have been of critical importance as tools to study and expand the knowledge of cardiac physiology and disease. For several years, cell culture techniques have been physiologically relevant to understand living organisms both structurally and functionally since they are thought to preserve as closely as possible the in vivo integrity and function of cardiac cells [1].

The study of the heart through cell culture has allowed the visualization of cellular structures and the study of physiological mechanisms such as intracellular Ca^2+^ homeostasis [2], electrical activity of the cells under experiment [3,4], ionic models [5], cellular contraction mechanisms [6], as well as cellular signaling mechanisms [3]. To emulate the extracellular environment in which the cells grow is of vital importance to understand these phenomena in different cell types [7]. More recently, the careful selection of the appropriate substrate on which the cells are cultivated has been proven to be critical, since the microenvironment may dictate cellular behavior [8]. The cardiac extracellular matrix (ECM) has the structural role of giving strength and support to the cardiac cells. The ECM is critical to maintaining the structural integrity of the heart and disruption of the matrix network results in alterations of the ventricular geometry. It has been reported that changes in ECM stiffness contribute to disruption of the coordination of myocardial excitation–contraction [9]. In particular, the use of microstructured platforms that simulate the mechanical properties of different cellular niches may significantly improve the quality of cell culture. Van Deel et al., in 2017, showed that rat cardiomyocytes cultured in polyacrylamide gels with different stiffnesses modified contractibility. A stiffer matrix (100 kPa) prevents cell shortening, shortening velocity, and relaxation velocity in cardiomyocytes [10]. It has been reported that the fate of mesenchymal stem cells cultured on soft matrices tends to the growth of neurons, stiffer matrices become myogenic, and comparatively rigid matrices prove to be osteogenic [11,12,13]. The contractile capacity of cardiomyocytes requires a substrate to which they can attach. Changes in the matrix to which they are attached can act on physiological properties such as the contractile force or ion conduction [13,14,15] enabling the study of phenomena such as reentrant arrhythmias and the functional consequences of cardiomyocyte–matrix interactions, and cardiac pathologies like the accumulation of ECM or fibrosis [16].

Among polymers used for microplatform applications besides polystyrene, one of common use is polymethyl methacrylate (PMMA). This material is transparent, films can be directly patterned using electron-beam lithography, and this plastic can be surface modified to introduce amine groups for DNA purification [17]. However, stiffness is not easily controllable, and its manufacture requires specialized equipment.

Polydimethylsiloxane (PDMS) is a low-density material with highly hydrophobic surface properties, low surface and bulk conductivity, contamination resistance, and long-term endurance [18]. PDMS is an off-the-shelf polymer of widespread use in the biomedical field, since all its interesting properties, including optical transparency and porosity to gases and ease of use in soft-lithography to replicate micromolds, make it a perfect candidate for complex cell culture platforms integrating several important features like micropatterns or fluidic microchannels. Another important favorable characteristic for cell culture is the possibility offered by the elastomer to readily tune its mechanical properties within a physiological range, from a few kPa up to a couple of MPa [19]. Ming Ni and colleagues review the properties of the cell culture surface as topography, protein adsorption, and adhesive interaction between the cell and substrate. They also describe different cell types cultivated on bioreactors and 3D variants of this substrate such as that of human fetal hepatocytes; liver cells, osteoblastic cells. Nanogrooved PDMS channels became suited to grow endothelium in linear vessels. PDMS has been successfully used to build microfluidic channel-based systems for cell culture. It has also been possible to culture mammalian embryos in microfluidics chips. However, none of these systems is completely equivalent to in vivo conditions, as it lacks cell–ECM interaction, cell–cell interaction, soluble factors, and mechanical forces in 3D [17].

As previously mentioned, the stiffness of cardiac cells in their in situ environment is very relevant, between 10 and 20 kPa, but it increases markedly when a heart attack occurs (35–60 kPa) [6]. The extracellular matrix stiffness plays a significant role in proliferation, migration, and even in cell differentiation [20], and these processes are linked to the process of mechanotransduction [21]. Controlling the stiffness of culture substrates is thus of critical importance when biomimetic cultures are sought to study cardiac cells and tissues.

In this work, we constructed PDMS microscaffolds of two different rigidities to find the optimal conditions of stiffness that mimic the ECM, through the evaluation of intracellular calcium signals. Establishing these conditions would indeed allow generating microscaffolds in order to study different phenomena like cardiac pathologies. PDMS microplates were made using different stiffness conditions, and calcium signals were recorded in chicken embryo cardiomyocytes after 24 h of culture, to determine how the substrate stiffness and substrate characteristics after UV radiation affected the adhesion and contractile activity of chick cardiomyocytes when studied using intracellular calcium signaling at a population level [22].

## 2. Materials and Methods

### 2.1. Manufacture of PDMS Microscaffolds

**Figure 1:**
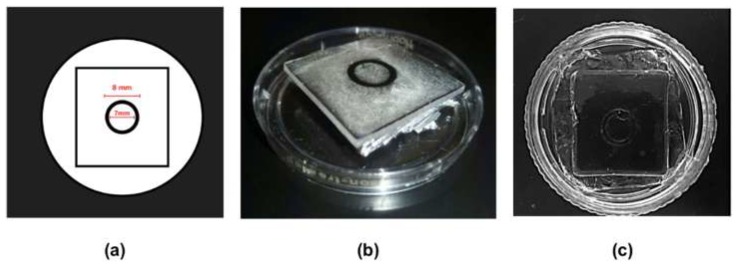
Fabrication of polydimethylsiloxane (PDMS) microscaffolds (**a**) Shrinky Dink^®^ mold design, (**b**) Shrinky Dink^®^ stamp adhered to the lid of a 3.5 mm Petri dish, (**c**) PDMS microscaffold replica.

PDMS microscaffolds were fabricated following a process reported in [23]. First, the molds for microfluidic channels were designed in free software (Inkscape 0.91). They were shaped as rings with an 8 mm external diameter and a 7 mm inner diameter (Figure 1a). Designs were printed onto a polystyrene thermoplastic sheet (Shrinky Dinks^®^, K&B Innovations, North Lake, WI, USA). Once printed, these micromolds were cleaned with 3% diluted isopropyl alcohol and acetone to better define the features by removing undesired ink around them. The printed polystyrene thermoplastic sheets were finally placed in an electric convection oven preheated to 175 °C; upon heating, the engravings shrunk by almost 60% and the ink shapes increased in height.

PDMS microscaffolds were then fabricated using soft lithography using the Shrinky Dinks^®^ molds. Two commercial silicone elastomer kits (Dow Corning) were combined for stiffness control, as reported in [19]. Indeed, by mixing Sylgard^®^ 184 (with a reported Young’s modulus of 1720 kPa) and Sylgard^®^ 527, (5 kPa) in different proportions, it is theoretically possible to obtain PDMS slabs with Young’s modulus from 5 kPa to 1.5 MPa.

Cardiomyocyte extracellular matrix stiffness is relatively low (10–20 kPa) [6]. The PDMS microscaffolds were fabricated by first preparing each PDMS form separately by mixing their respective prepolymers and curing agents in a 10:1 w/w proportion and then combining them in a 1:10 and a 1:30 (184:527) ratio. The PDMS mixture was thoroughly mixed for 5 min and degassed under vacuum for 15 min. The solution was poured into a 35 mm diameter Petri dish; the Shrinky Dinks stamp was fixed at the top of the Petri dish (Figure 1b). The PDMS was then cured at 60 °C for 48 h in a preheated convection oven and then released from the stamp after a 30 min cooling down to form a PDMS culture platform (Figure 1c). The microscaffold stiffness was calculated from micro-indentation measurements using the Hertz model (FemtoTools MTA-02 system, with a sphere of 50 micron radius) and the values of Young’s moduli are reported in Table 1. Before seeding the cells, PDMS platforms were cleaned with ethanol and irradiated with UV light (type C) for 30 min.

Measurements of the mechanical properties of the platforms were performed using a FemtoTools FT-MTA02 micro-indentation system (FemtoTools, Buchs, Switzerland). All samples showed an elastic behavior, and the analysis using a Hertz model resulted in an average Young’s modulus of 15 and 25 kPa for the softest and the stiffest sample, respectively, very close to what was reported previously [19]. Although a lower value was expected for the 1:30 ratio, this very soft material was very difficult to measure several times due to its very high adhesion to the fragile tip of our system and could provoke breakage.

### 2.2. Cell Culture

Cell cultures were prepared according to previously established procedures [24]. Ventricular cells were isolated from chick embryos hearts after seven to eight days of incubation in ovo. The ventricles were dissociated by an enzymatic procedure and plated at densities of 4 × 10^4^ cells/mL. The cell culture was incubated overnight in a cell culture medium (M199; Gibco Life Technologies, Grand Island, NY, USA) supplemented with 10% horse serum (Gibco Life Technologies), 5% fetal bovine serum (Gibco Life Technologies), 17 mM HEPES (Sigma), and gentamicin (Gibco Life Technologies) at 37 °C, CO_2_ 5%. After the enzymatic dissociation, the viability percentage was determined by trypan blue staining, later, cells were seeded and cultured for 24 h, the living cells adhered to the substrate and form a monolayer, the dead cells remained in the supernatant, which was eliminated when the culture medium was replaced by Hank’s solution. 

### 2.3. Intracellular Calcium Imaging

**Figure 2:**
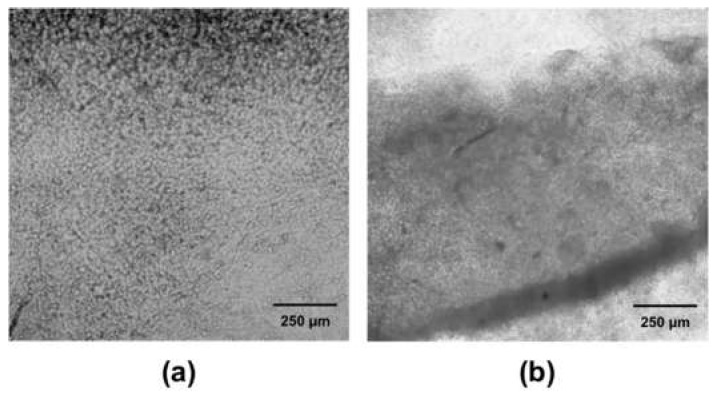
Bright-field micrograph of embryonic chicken cardiomyocytes cultured on a PDMS microscaffold (soft PDMS). (**a**) Cardiomyocytes in a monolayer attached to the center of the ring in the soft PDMS. (**b**) Cardiomyocytes in a monolayer attached to the ring channel.

For the experiments, cells were incubated 30 min at 37 °C, 5% CO_2_ with 22 µM cell-permeable calcium sensor calcium green-1 (Molecular Probes®; Eugene, OR, USA) at a final concentration of 4 µM (prepared from a 4 mM stock solution in DMSO with 0.5% pluronic acid (F-127 Sigma)) in Hank’s solution (in mM: 125 NaCl, 0.9 KCl, 3.6 NaHCO_3_, 0.3 Na_2_HPO_4_, 0.4 KH_2_PO_4_, 0.5 MgCl_2_, 0.4 MgSO_4_, 10 glucose, 2.9 sucrose, 9.9 HEPES, and 2.2 CaCl_2_, pH 7.2) Once washed, cells were transferred to the fluorescence stereo microscope stage with controlled temperature (36 ± 0.5 °C). Cell cultures were reviewed with a Leica MZ75 stereomicroscope (Leica, Wetzlar, Germany), and fluorescent images were acquired with a Leica DFC360FX camera (Leica, Wetzlar, Germany) under protocols written in Micro-Manager 1.4; sequences of 2000 images (80 ms exposure, 40 ms interval) were taken. For the processing and analysis of the intracellular calcium fluctuations associated with the spontaneous contractile activity of the embryonic cardiac cells, we employed ImageJ (NIH, USA) and Igor Pro (WaveMetrics Inc., Lake Oswego, OR, USA) software. Since calcium green-1 is a single-wavelength dye, its fluorescence emission is a function of dye concentration, illumination pathway, and intracellular [Ca^2+^]. According to our experimental conditions, only fluorescence changes related to [Ca^2+^]_I_ fluctuations were significant. Transmitted light images were acquired with an inverted fluorescence microscope ImageXpress XL (Molecular Devices) after intracellular Ca^2+^ experiment was recorded (Figure 2). 

### 2.4. Cell Viability

Cell viability in the monolayers was measured through the Calcein–Propidium Iodide Assay. After 48 h of culture, the cell culture medium was replaced by Hank’s solution and cells were incubated with calcein green AM 1 µM Molecular Probes^®^; Eugene, OR, USA) and propidium iodide 1 µM (Molecular Probes^®^; Eugene, OR, USA) for 15 min. Two-channel images (calcein–AM/propidium iodide (PI)) were acquired, where green (488 nm) and red (545 nm) fluorescent cells represent live and dead cells, respectively. Transmitted light images and viability images were acquired with an inverted fluorescence microscope ImageXpress XL (Molecular Devices) (*n* = 9). 

### 2.5. Data Analysis

To identify intracellular calcium signals, “standard deviation images” (SD) were obtained from recorded movies (standard deviation algorithm of ImageJ: stack projection functions). In SD images, areas of high pixel values correspond to cells with large intracellular calcium signal changes, and areas with low pixel values correspond to regions that were less active or remained silent. Pixel intensity values were color coded for illustration purposes. Regions of interest (ROIs) corresponding to areas with activity were drawn on the SD image. Using this approach, records from the whole platform can be readily obtained. A convenient way to represent this large amount of data is the multicell ∆F (t) plot. These plots were generated with Igor Pro 5.03 macros (WaveMetrics Inc., Lake Oswego, OR, USA). Here, ordinates represent ROIs number (one ROI per row), the time is represented in the abscissa, and fluorescence intensity values are color coded. From these plots, traces from the ROIs can be extracted for analysis [25]. To assess the percentage of adherence of the cells to the scaffold, the ROIs were identified (both in the ring channel and in the flat part) and their contour was drawn on the SD images, the area was measured using National Institutes of Health ImageJ routines (“set scale”: For conversion of pixels to microns; “set measure”; and “area”). To measure the cell viability, the percentage area covered by the live cells stained with green calcein and the dead cells stained with propidium iodide (IP) was calculated through the Analyze Particles routine of the ImageJ program (National Institutes of Health). To verify the reliability of the staining with both dyes, at the end of the experiment, cells were treated with a KCl solution 3 M, which induced cell death.

## 3. Results

### 3.1. Biocompatibility, Adhesion, and Monolayer Formation.

**Figure 3:**
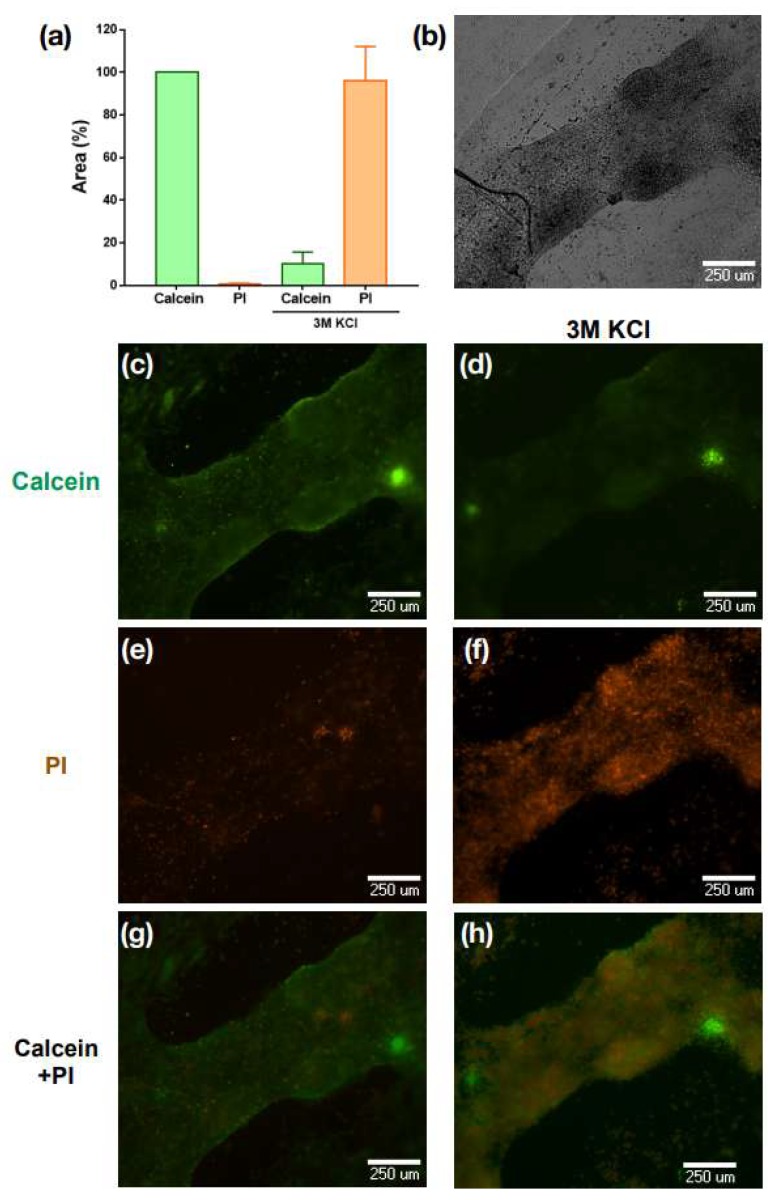
Viability and biocompatibility of PDMS microscaffolds and cardiac cells. (**a**) Graph of the percentage of live cell area vs. dead cells and after application of 3 M KCl, (**b**) monolayer micrograph of cardiomyocytes in brightfield. Live cardiomyocytes labeled with calcein after 24 h of culture (**c**) and after being exposed to KCL 3 M (**d**). Dead cardiomyocytes labeled with propidium iodide after 24 h of culture (**e**) and after being exposed to KCL 3 M (**f**). (**g**,**h**) are the superposition of (c,e) and (d,f), respectively.

The percentage of living cells after the dissociation and before being seeded were verified through trypan blue staining. We obtained viability of 92.68% ± 5.02% (mean + SE), *n* = 5. Normally, cell culture plates are coated with collagen or Poly-l-lysine as extracellular matrix to improve the initial adhesion; nevertheless, in our case, radiation with UV light for 30 min during sterilization was a sufficient surface modification to attach cardiomyocytes to PDMS. No type of extracellular matrix was needed for adhesion, adding to the simplicity of the use of our scaffold for our application. Indeed, the use of UV irradiation was reported to hydrolyze and replace methyl side chains by OH hydroxyl groups in PDMS, thus leading to a hydrophilic, adhesion-promoting surface. The cells were seeded and cultured in sterile conditions for 24 h on the microplatforms to form a monolayer on the flat substrate (inner part of the ring) (Figure 2a) and a thicker layer in the ring channel, which has a depth of 80 microns (Figure 2a). One of the most suitable measurements for cell viability in cardiomyocytes is a contractile response [26]; in our case, embryonic cardiomyocytes developed spontaneous activity and contractibility, therefore, to assess microplates viability and biocompatibility with the cells after being cultured on the PDMS, the contractile activity was recorded. Supplementary videos 1 and 2 (Videos S1 and S2) show cardiomyocyte contractility after 24 h of culture. Viability and biocompatibility were verified when monolayers were labeled with calcein-AM, green cells represent live cells (Figure 3b), and dead cells are with propidium iodide (PI) and are shown in orange (Figure 3c). The percentage of area was plotted normalizing the area covered by cells stained with PI, calcein + KCl 3 M and PI + KCl 3 M with respect to the area covered by cells stained with calcein, which was considered as 100% of the stained area. 

Figure 3a shows that the proportion of living cells is greater with respect to dead cells, this proportion was altered after adding 3 M of KCl. Here an increase in the area corresponding to the PI staining is observed, and therefore an increase in the number of dead cells is observed, which is also consistent with the representative images shown in Figure 3c–h. This result demonstrates the biocompatibility of the PDMS microscaffolds, as well as the viability of the cells grown on them.

### 3.2. Calcium Signaling in Different Substrates

It is well known that calcium (Ca^2+^) is essential for cardiomyocyte function since it is responsible for the excitation–contraction coupling (EC coupling). EC coupling is the process that links electrical signals with the contractile protein machinery in cardiomyocytes [27]. Therefore, calcium signaling is an indirect measurement of electrical activity and cells contractility. At a population level, it is necessary to preserve as closely as possible the in vivo integrity, properties, and function of the cardiomyocytes. For the optimal functioning of the heart, the electrical, contractile, and intracellular calcium activity of the cardiomyocytes has to be coordinated; therefore, the matrix of the cardiomyocytes has to allow them to work in coordination. 

**Figure 4:**
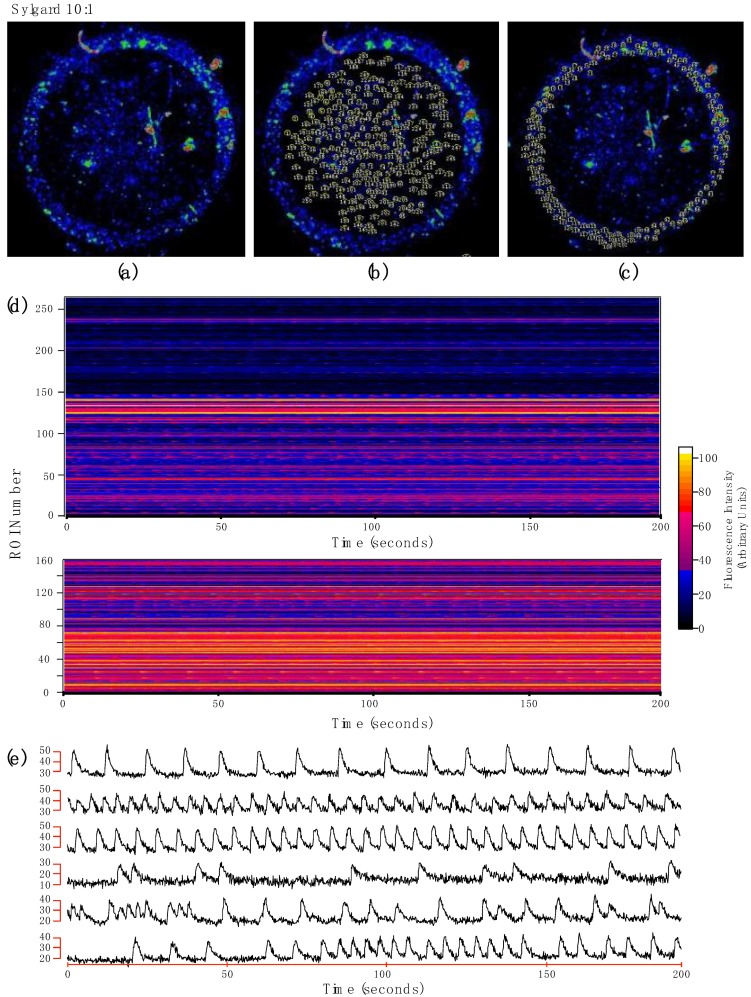
Embryonic cardiomyocytes cultured in stiff PDMS (**a**) SD image corresponding to the cardiomyocytes cultured in stiff PDMS. Movies were acquired with a stereoscopic microscope (2X). Fluorescence intensity of Ca^2+^ signals of embryonic cardiomyocytes labeled with calcium green-1 grown on a PDMS microscaffold of proportion 1:10. Selection of regions of interest (ROI) from the optical recordings for the study over 200 seconds in the center of the ring (**b**) ring channel (**c**). (**d**) Multicell (raster chart) summarizes the variations of Ca^2+^ activity over the time of 269 ROIs (upper panel) and 150 ROIs (lower panel). The ordinate axis represents the ROI number of the region marked in (**b**,**c**) (one ROI per row). The recording time is plotted on the abscissas, the intensity of the fluorescence for each region shown according to the color scale on the right side, the intensity is in arbitrary units (AU). (**e**) Representative examples of the intracellular calcium traces extracted from the ROIs shown in (**b**,**c**). The traces show the differences in intensity as well as the asynchrony of the calcium responses.

Figure 4 illustrates Ca^2+^ signals recorded from cardiomyocytes cultured on stiff PDMS. With this approach, Ca^2+^ activity can be recorded at the population level. Pixel intensity values were color coded for illustration purposes, areas with high pixel values correspond to cells with large modifications of intracellular calcium signal (white), areas with low pixel values correspond to areas with less activity (black). Since the flat substrate seems more porous than the ring channel here, we tested two different platform sections, the flat substrate (ring center) where only two layers of cells were formed, and the ring channel wherein cell ensembles formed microtissue, with the aim to observe if adhesion is affected by a higher cell density. Figure 4a shows the standard deviation image of the cardiomyocytes cultured on stiff PDMS. ROIs (regions of interest) were drawn in the SD image in the flat substrate (Figure 4b) and the ring channel (Figure 4c), to verify if the intracellular Ca^2+^ activity is homogeneous at the center of the ring as in the ring channel. On stiff PDMS, cardiomyocytes did not adhere uniformly to the platform, they did not form a monolayer, on the contrary, they formed aggregates of cells on the flat substrate as well as inside the ring channel. Although the cellular aggregates show intracellular calcium activity, it is not synchronous (Video S3). Figure 4c,d shows the traces of calcium activity of ROIs in different areas.

The y-axis shows the fluorescence intensity in arbitrary units (AU) and the x-axis time (200 s). Figure 4e shows representative examples of the intracellular calcium activity of ROIs taken from Figure 4b,c, over 200ms, we observed that some traces show fluorescence intensities ranging from 10 to 40 AU and others from 40 to 70 AU and calcium transients do not occur at the same time in the different tracings, demonstrating the absence of a population coupling. We did not find coordinated Ca^2+^ activity along the platform. Cells do not cover the whole scaffold, form aggregates which cover 45% of the micro-scaffold. Even though a higher density of cells is seen in the channel, they did not form a continuous layer; on the contrary, they formed groups of cells that do not connect. In Figure 3d, each line on the y-axis represents an ROI, and the different colors show the changes in the intensity of fluorescence. In the upper panel, the fluorescence intensities remain mainly in the range of blue and purple, which means that, in those areas, there is less fluorescence intensity; unlike in the lower panel, a higher intensity of fluorescence is observed, since the colors are mostly in the range of orange, pink, and red. In this type of graphics when the calcium response is synchronous, the color changes occur at the same time, (independently of the intensity of the fluorescence), however in the two panels of the Figure 4d graph the synchronous activity is not observed.

**Figure 5:**
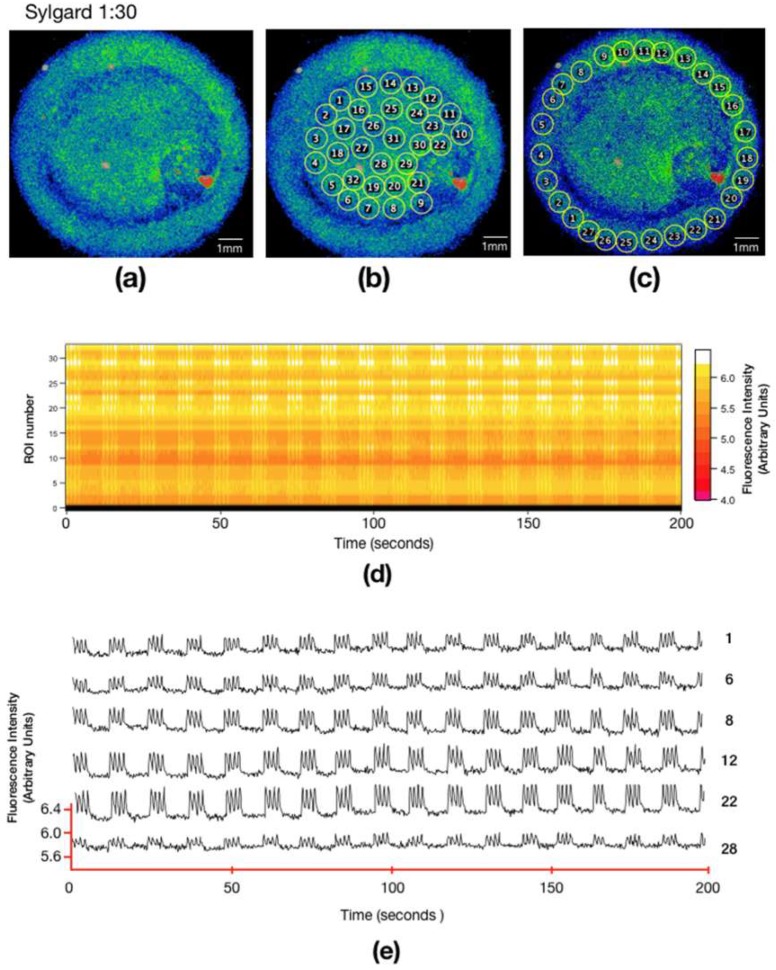
Embryonic cardiomyocytes cultured in soft PDMS (**a**) SD image corresponding to the cardiomyocytes cultured on proportion 1:30 of PDMS combination. Selection of regions of interest (ROI) from the optical recordings for the study over 200 seconds in the center of the ring (**b**) and in the ring channel (**c**). (**d**) Multicell (raster chart) summarizes the variations of Ca^2+^ activity over the time of 40 ROIs. The ordinate axis represents the ROI number of the region marked in (b) (one ROI per row). The recording time is plotted on the abscissas, the intensity of the fluorescence for each region shown according to the color scale on the right side, the intensity is in arbitrary units (AU). (**e**) Activity profile from calcium fluorescence of six ROIs from the center of the ring.

On soft PDMS, the cardiomyocytes formed a uniform monolayer of cells throughout the entire microplate (Figure 5a), cells covered the whole scaffold. Similar to the stiff PDMS microscaffold, the ROIs were drawn both in the center of the ring (Figure 5b) and in the channel (Figure 5c). The calcium activity of each ROI was measured over 200 s, traces of different areas are shown in Figure 5e. It can be observed in the multicell plot (Figure 5d), and in the individual traces (Figure 5e), that the calcium activity is synchronous (Video S4). Even though there are areas in which the fluorescence ranges are of lower intensity, the cells elicit calcium transients at the same time. Soft PDMS has Young’s modulus of 25.76 ± 6.21 kPa (Table 1), which is very similar to the Young’s modulus of the ECM, which has been reported between 10 and 20 kPa [6]. In the multicell graph (Figure 5d), the synchronicity of the calcium signals is observed, the white vertical lines imply that calcium changes are occurring at the same time in all the ROIs labeled. Figure 5 shows that the cells have better adhesion compared to the stiff PDMS since the scaffold forms monolayers and synchronizes calcium activity.

## 4. Discussion

Entire hearts or cultured cardiomyocytes from chick embryos have been used to study many morphological [28], biochemical [29], toxicological [30], and electrophysiological [31,32] properties of the developing heart. A clear advantage of this experimental model is that the excitable and contractile properties of chick cardiomyocytes in culture are very homogeneous and stable, in our case, this model allows us to study the properties of the extracellular matrix on intracellular calcium activity. Many research groups are working on the differentiation of human-induced pluripotent stem cells (iPSCs) into cardiac cells since they are a promising cell source for cardiac tissue engineering and cell-based therapies for heart repair because they can be expanded in vitro and differentiated into most cardiovascular cell types, including cardiomyocytes [33]. Although iPSCs could be considered as a potentially unlimited source for the generation of cardiomyocytes, the current protocols for derivation present some challenges, including variability in somatic cell sources and inconsistencies in cardiac differentiation efficiency [30]. It has been observed that these cells exhibited immature calcium-handling properties and incompletely developed sarcoplasmic reticulum. Also, mixed action potential recordings are found which correspond to identified nodal-, atrial-, and ventricular-like phenotypes among the dissociated cells; with the ventricular-like phenotype being predominant [34,35]. Cardiomyocytes generated from iPSCs are typically developmentally immature in comparison to their adult counterparts [34]. Then we can consider that embryonic cardiac cell preparation is still a very relevant model to study cardiac physiology and pathologies.

Recent in vitro studies have shown that alterations of the elastic modulus of the substrate where cells are grown affect the proliferation rates of vascular smooth muscle cells, cell association, tissue formation, and differentiation of mesenchymal stem cells into myocytes and other cell types. These responses tend to approximate normal in vivo behavior more closely when the substrate stiffness is near that of the native extracellular matrix [13]. Also, it has been shown that substrate stiffness does affect force generation, with maximal force generation on substrates of 10 kPa, near the stiffness of native myocardium [13].

In the myocardium, tissue elasticity shows significant regional variation during heart disease [36,37,38,39,40]. Specifically, Berry et al. [41] observed that ischemic areas of rat myocardium showed a large increase as a function of the substrate elastic modulus, when comparing a physiological modulus of approximately 10–20 kPa to a greater modulus of 50 kPa. However, the effects of alterations in the substrate elastic modulus on the auto-organization of cell monolayers had not been described.

Our results indicate that the cell culture substrate stiffness induces definite changes in adhesion and organizing properties of the connectivity between cardiomyocytes. Firstly, on our stiffness-controlled PDMS preparations, the cells attach to the substrate without the addition of any other adherent or extracellular component, this helps us to replicate the native environmental conditions of the cells. It is known that UV irradiation for 30 min promoted cell adhesion as the methyl side chains going through hydrolysis under UV light result in hydroxyl-terminated (silanol) groups at the surface rendering it hydrophilic. UV is reported to be less aggressive than plasma exposure, avoiding a stiffness modification at the surface [22]. Combining PDMS Sylgard 184 and 527 in a 1:10 ratio, corresponding to a Young’s modulus greater than what is usually reported in vivo, the cells did not adhere to the substrate, and they had to form spherical aggregates. Conversely, with a more physiological stiffness of a 1:30 ratio, the cardiomyocytes adhere, extend, and constitute well-connected layers that display synchronized activity [42].

Another approach has been to develop nanofiber scaffolds made by electrospinning which have useful characteristics in regenerative medicine such as physical properties; nanometric diameters; high surface/volume ratio close to those of living tissues; adhesion and growth of living cells. Several biocompatible and biodegradable polymers such as those based on poly(L-lactic acid), poly(Ɛ-caprolactone), poly(glycerol sebacate), poly(polyphosphazene), chitosan, and collagen, have been studied as scaffolds for tissue engineering due to their physicochemical and biological properties as well as simple processing [43].

PDMS is not always the best choice of material for cell culture, and especially in mechanobiology, as the stiffness ranges available are not suited for all tissues or cells, and it may also be a difficult material to work with, because of the potential harm of its oligomers left after badly controlled curing. In this work, the cell culture platforms were carefully cured for 48 h at 60 °C in a convection oven and washed several times with Phosphate Buffered Saline (PBS) after curing in order to avoid any possible poisoning of cells by oligomers. Indeed, as the structures are aimed at being eventually integrated into microfluidic chips, PDMS is an excellent candidate because of its ease of use in micropatterning and soft-lithography. In our case, it allowed us to obtain a micro-scaffold that contains the characteristics of the ECM, which is convenient for the study of cardiomyocytes and cardiac tissue. This type of microscaffold could help us study phenomena such as EC coupling or cardiac arrhythmias. Our observations are relevant to current controversies concerning the best methods or procedures to obtain implantable patchworks of cardiac tissue which can mimic its intrinsic properties and native characteristics. Likewise, the activity pattern revealed by the variations in calcium fluorescence is reliable to track the functional organization that the cell layers achieve. For example, the emergence of synchronized activity or rotor formation that can initiate or maintain cardiac arrhythmias can be better evaluated in different substrates before designing a replacement or patch tissue.

## 5. Conclusions

In this study, we found that the soft PDMS with Young’s modulus of 25.76 + 6.21 kPa was the one in which the cells best adhered since they covered the whole scaffold, forming a monolayer and showing synchronous calcium-related activity, which is essential for the study of cardiac tissue. This type of scaffolding can be a good model for studying the behavior of cardiomyocytes to test drugs of interest or to consider them as isolated pacemakers. The soft scaffold of PDMS presents a stiffness similar to that of the native extracellular matrix of the cardiomyocytes, which makes it an excellent tool for cardiomyocyte culture. Additionally, in this scaffold, calcium activity is uniform and cells form a continuous monolayer. These characteristics can be used to study the behavior of cardiac tissue; in particular, if cells are cultured inside the ring channel, their behavior may also help study phenomena such as reentrant arrhythmias.

## Figures and Tables

**Table 1:** Microscaffold stiffness measurements. Mean + SD (three samples, three locations, except for 527 only and 30:1, as the viscosity, only allowed for three measurements in total).

Mixture	Ratio	Young’s modulus (kPa) (mean ± SD)
527 only	0	15.16 ± 4.45
184 only	1	1100 ± 77.45
30:1	0.03	25.76 ± 6.21
10:1	0.1	54.87 ± 3.62

## Supplementary Materials

The following are available online at https://www.mdpi.com/2313-7673/4/2/33/s1, Video S1: Cardiomyocytes cultured on the microplatforms to form a monolayer on the flat substrate in brightfield. Two thousand images were taken with an exposition time of 127 ms. Video S2: Cardiomyocytes cultured on the microplatforms to form a thicker layer in the ring channel substrate in brightfield. Two thousand images were taken with an exposition time of 127 ms. Video S3: Intracellular calcium signals in cardiomyocytes cultured in stiff PDMS. Two thousand images were taken with an exposition time of 127 ms. Video S4: Intracellular calcium signals in cardiomyocytes cultured on soft PDMS. Two thousand images were taken with an exposition time of 127 ms.
